# A Study on the Evolution of Intermetallic Phase Microstructure and High-Temperature Creep Behavior in Mg–8.0Al–1.0Nd–1.5Gd–Mn Alloys

**DOI:** 10.3390/ma19122681

**Published:** 2026-06-22

**Authors:** Jiandong Yang, Wuxiao Wang, Liwen Zhang, Peng Zhou, Tianjun Bian

**Affiliations:** 1 Department of Aeronautical Materials Engineering, Xi’an Aeronautical Polytechnic Institute, Xi’an 710089, China; yangjiandong7c@163.com (J.Y.); pzhou1975@163.com (P.Z.); btj329491@163.com (T.B.); 2 School of Material Science and Engineering, Xi’an University of Technology, Xi’an 710048, China; wangwx@xaut.edu.cn

**Keywords:** Mg–Al–RE alloy, intermetallic compounds, compression creep, dislocation slipping, mechanical properties

## Abstract

The effects of Mn/RE (Nd, Gd) multi-modification on the microstructure and high-temperature compressive creep properties of Mg–8.0Al alloys were investigated. The dominant intermetallic phases in the as-cast microstructure are *β*-Mg_17_4Al_12_, Al_2_(Gd,Nd), Al_11_(Gd,Nd)_3_, Al_8_(Gd,Nd)Mn_4_, and Al_10_Mn_2_(Gd,Nd). The detailed structures of various intermetallics were revealed by TEM; the results indicate that Mn addition promotes grain refinement and facilitates the precipitation of lath-shaped and spherical *β*-Mg_17_Al_12_ in as-cast Mg–Al–RE alloys, resulting in increases in the tensile strength and elongation of the 1.0Mn alloy by 26.5% and 92.1%, respectively. Additionally, thermally stable micron-scale Al_8_(Gd,Nd)Mn_4_ and Al_12_(Gd,Nd)_2_Mn_5_, along with dynamically precipitated spherical nano-sized AlGd and AlNd particles in the α-Mg matrix, were innovatively observed in compression-crept specimens tested at 200 °C and 60 MPa; these phases play a key role in improving high-temperature creep resistance. A significant finding is that excessive Mn addition deteriorates creep performance, which is attributed to excessive grain refinement and the consequent increase in the contribution of grain boundary sliding during creep. However, the negative effect of grain boundary sliding—caused by grain refinement—on creep performance can be balanced by the strengthening effect of Al–Mn–Gd phases and the dynamic precipitation of nanoscale Al–RE particles. This paper provides new insights for designing Mg–Al–Nd–Gd–Mn alloys with both excellent high-temperature creep resistance and significantly enhanced mechanical properties.

## 1. Introduction

The optimization of Mg–Al–RE-based alloys for improved mechanical properties and creep resistance is an urgent requirement for lightweighting in the automotive industry [1,2,3]. To achieve a balance between strength and ductility while enhancing their suitability for service at elevated temperatures—such as in components of automotive transmission systems—an effective strategy is to add RE elements to the Mg–Al alloys [3,4,5]. The excellent creep resistance of Mg–Al–RE (Nd, Gd) alloys is attributed to thermally stable intermetallics such as Al_2_RE and Al_11_RE_3_ [6,7,8,9,10]. However, pursuing high strength and high creep resistance often comes at the expense of reduced ductility, resulting in most Mg–Al–RE alloys exhibiting elongation values between 3% and 6%. Therefore, simultaneously improving both creep performance and ductility remains a key challenge in developing high-performance Mg–Al alloys. Research has shown that incorporating Mn into Mg–Al–RE alloys can effectively refine the α-Mg matrix grain size and suppress the formation of coarse eutectic *β*-Mg_17_Al_12_—which adversely affects mechanical properties—thereby enhancing both strength and ductility. However, an important issue arises: refining the Mg matrix grains increases the number of grain boundaries, resulting in a higher proportion of grain boundary sliding (GBS) during creep and thus more severe creep deformation. This gives rise to a conflict between achieving high plasticity and maintaining excellent creep resistance. Studies have indicated that adding Mn to Mg–Al–RE alloys promotes the precipitation of Al–Mn–RE intermetallic phases, such as Al_8_GdMn_4_ [11,12,13], Al_12_RE_2_Mn_5_ and Al_10_RE_2_Mn_7_ [14], which contribute to strengthening via second-phase particle strengthening. It is experimentally confirmed that Mg–9Al–0.3Ca–0.2Mn (wt. %) with a minor content of Mn elements simultaneously increases strength and ductility [15]. A newly developed Mg–4Al–3La–0.3Mn (wt. %) alloy exhibits excellent tensile properties, which is attributed to the grain refinement caused by various intermetallic compounds [16]. It has been demonstrated that Al_8_Mn_5_ particles are ineffective as heterogeneous nucleation sites for refining *α*-Mg grains during solidification [17,18]. According to the Hall–Petch relationship, grain refinement is the primary strategy for enhancing the strength of Mn-containing Mg–Al alloys [19,20,21]. A high density of AlMnGd precipitates (approximately 2~10 nm) impedes dislocation motion in the Mg–3.5RE(La,Ce,Nd)1.5Gd–Mn–Al alloy, playing a major role in improving creep resistance [22]. Additionally, nano-sized Al-Mn and *α*-Mn phases dynamically precipitate in crept specimens and hinder dislocation motion for improving the creep resistance [23,24,25,26]. Most current research focuses on how Mn addition influences the mechanical properties of Mg–Al–RE alloys, whereas the thermal stability of these intermetallic compounds and their impact on high-temperature creep behavior remain poorly understood. Moreover, the relatively high solubility of Mn in Mg affects the characteristics of intermetallic compound precipitation during the solidification of Mg–Al–RE alloys; however, the exact types, morphologies, and volume fractions of the precipitated intermetallic phases are still not fully clarified.

In this paper, Mg–8.0Al–1.0Nd–1.5Gd–*x*Mn (*x* = 0.3, 0.5, 1.0 wt. %) alloys were designed, and various Mn-containing intermetallic phases were thoroughly investigated. The relationship between the beneficial role of multi-component intermetallics in enhancing creep resistance and the detrimental effect of grain refinement on creep performance requires further investigation. This paper focuses on the thermal stability of Al–Mn–RE ternary phases formed under gravity casting conditions during high-temperature creep as well as on the types of dynamically precipitated phases during creep and their contributions to improved creep resistance. By tailoring the precipitated phase distribution in the magnesium alloy, both the elongation and high-temperature creep resistance are simultaneously enhanced.

## 2. Experimental Material and Procedures

The specific alloys were prepared from magnesium ingot (99.99 wt. %), aluminum ingot (99.99 wt. %), and Mg–30Gd, Mg–30Nd and Mg–15Mn (wt. %) master alloys. Magnesium and aluminum ingots were initially melted in a resistance furnace at 710 °C under a protective atmosphere of Ar. the intermediate alloys were added, which was followed by 30 min of soaking to ensure complete melting. Homogenization of the alloy chemical composition was achieved through mechanical stirring, and the ingots were fabricated by gravity casting. The Mg–8.0Al–1.0Nd–1.5Gd–*x*Mn (*x* = 0.0, 0.3, 0.5, 1.0 wt. %) alloys with different Mn contents were abbreviated as the 0.0Mn, 0.3Mn, 0.5Mn, and 1.0Mn alloys.

The ingots were processed into cylindrical specimens for compression creep testing; the specimens were subjected to a constant load of 60 MPa/200 °C for 100 h. The temperature control accuracy was ±1 °C, and the displacement sensor with an accuracy of ±0.001 mm was used to measure the deformation of the crept samples. The creep rate was calculated according to creep strain–time curves. Tensile tests were conducted on a testing machine (HT-2040) (Hung Ta Instrument Co., Ltd., Taichung City, Taiwan) at ambient temperature with a tensile rate of 0.5 mm/min. The average value of three tensile tests was taken for each specimen, and the deformation displacement was measured using an extensometer.

The microstructure was characterized via X-ray diffraction (XRD) with Cu Kα radiation (λ = 1.5418 Å) and a JEOL JSM-6700F (JEOL Ltd., Akishima, Tokyo, Japan) scanning electron microscope (SEM) equipped with an electron backscatter diffraction (EBSD). The samples were mechanically polished to a final thickness of 100 μm, and foils with a diameter of 3 mm were prepared for transmission electron microscopy (TEM) testing, which was followed by ion-beam thinning at 4 keV and a tilt angle of 4°. TEM was carried out on a JEM-2100F (JEOL Ltd., Akishima, Tokyo, Japan) microscope equipped with energy-dispersive X-ray spectroscopy (EDS), and high-resolution transmission electron microscopy (HRTEM) imaging was conducted at 200 kV. The HRTEM images were converted via Fast Fourier Transform (FFT) and Inverse Fast Transform (IFFT) functions in DigitalMicrograph (DM, GMS 1.x).

## 3. Results

### 3.1. Microstructures

**Figure 1:**
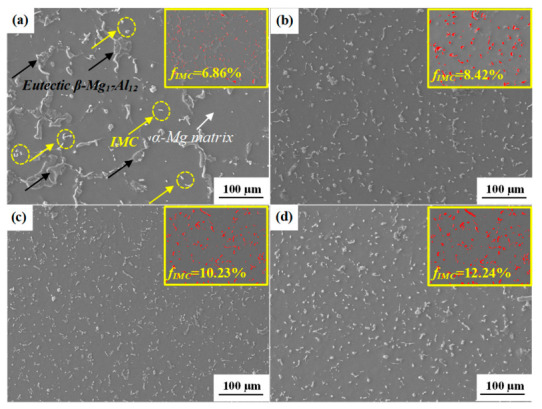
Microstructure of as-cast Mg–8.0Al–1.0Nd–1.5Gd–*x*Mn alloys: (**a**) 0.0Mn; (**b**) 0.3Mn; (**c**) 0.5Mn; (**d**) 1.0Mn.

Figure 1a–d show the SEM images of the as-cast Mg–8.0Al–1.0Nd–1.5Gd–*x*Mn (*x* = 0.0, 0.3, 0.5, 1.0 wt. %) alloys fabricated by gravity casting. Mn addition significantly refines the cellular microstructure of the Mg–8.0Al–1.0Nd–1.5Gd–Mn alloy. As the Mn content increases from 0.3 to 1.0 wt. %, the continuous, island-like eutectic *β*-Mg_17_Al_12_ phase transforms into fine, discrete eutectic particles. At the same time, the number of dispersed intermetallic compound particles (abbreviated as IMCs) has significantly increased, and the volume fraction of intermetallic compounds (*f*_IMC_) increased from 6.86% to 12.24% (*f*_IMC_ was calculated using Image-Pro Plus 6.0).

**Figure 2:**
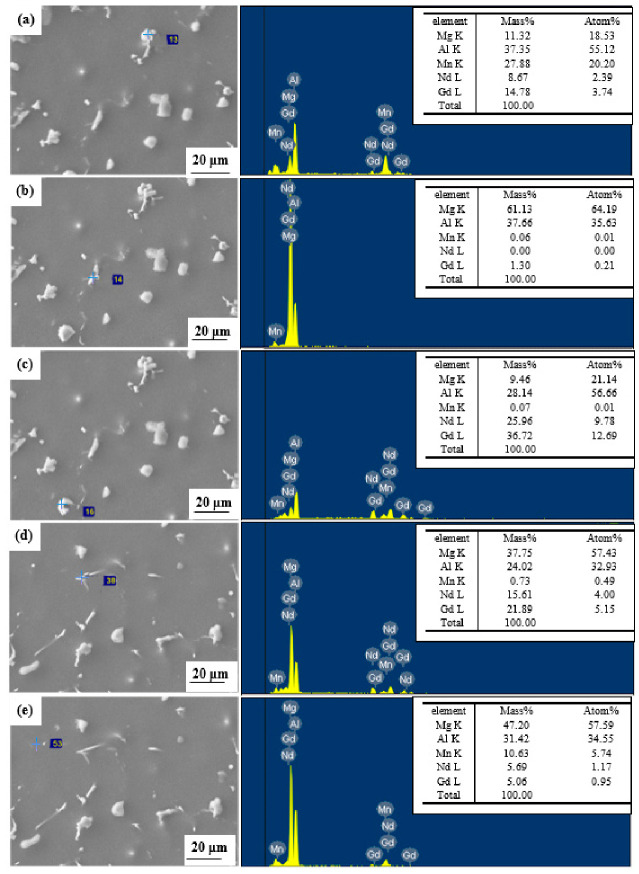
The representative EDS patterns for diversified intermetallic compounds of Mg–8.0Al–1.0Nd–1.5Gd–1.0Mn alloy: (**a**) Al_8_(Gd,Nd)Mn_4_; (**b**) *β*-Mg_17_Al_12_; (**c**) Al_2_(Gd,Nd); (**d**) Al_11_(Gd,Nd)_3_; (**e**) Al_10_Mn_2_(Gd,Nd).

**Figure 3:**
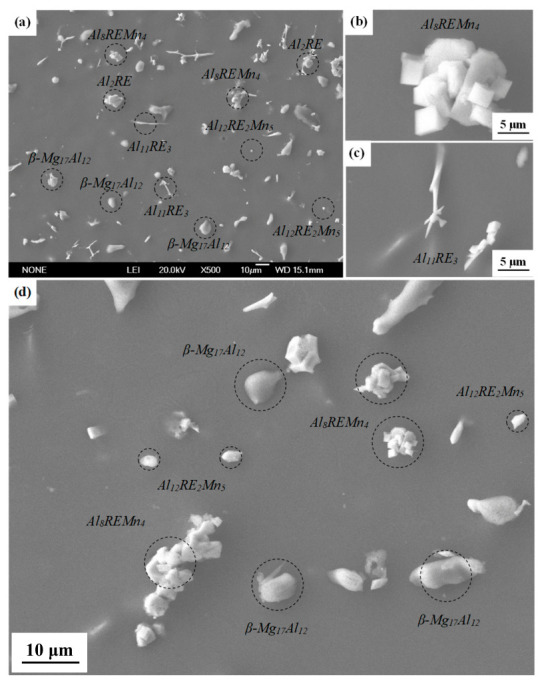
(**a**) Microstructure of 1.0Mn (wt. %) alloy; (**b**) agglomerated polyhedral block phase; (**c**) acicular phase; (**d**) corresponding magnified image of (**a**).

Figure 2 presents EDS quantitative analysis of precipitated phases with different morphologies in the Mg–8.0Al–1.0Nd–1.5Gd–1.0Mn alloy; the high Mg content detected in the EDS spectra of the precipitates is likely due to the signal contribution from the surrounding *α*-Mg matrix. It can be inferred that the polygonal block-like phase, acicular phase, short rod-shaped phase, polyhedral block-like phase, and fine granular phase correspond to Al_2_(Gd,Nd), Al_11_(Gd,Nd)_3_, *β*-Mg_17_Al_12_, Al_8_(Gd,Nd)Mn_4,_ and Al_10_Mn_2_(Gd,Nd), respectively (magnified images are shown in Figure 3a–d).

It can be seen that the initial semi-continuous reticular eutectic *β*-Mg_17_Al_12_ (~100 μm) particles are completely transformed into divorced eutectic short rod-shaped and spherical particles (5–15 μm) in the Mn-added alloys, confirming that the combined addition of Mn exerts a remarkable cell-refinement effect on the eutectic *β*-Mg_17_Al_12_ distributed at grain boundaries. The Al_8_(Gd,Nd)Mn_4_ phase is morphologically very similar to the reported Al_8_Mn_5_ in AZ91D alloy; thus, it is inferred that Al_8_(Gd,Nd)Mn_4_ may be transformed from Al_8_Mn_5_ [27]. As the Mn content increases from 0.3 to 1.0 wt. %, Al_8_(Gd,Nd)Mn_4_ gradually aggregates; simultaneously, fine particles of Al_12_(Gd,Nd)_2_Mn_5_ with a diameter of approximately 1.0 μm are dispersed in the *α*-Mg matrix, whereas acicular Al_11_(Gd,Nd)_3_, Al_2_(Gd,Nd), and Al_8_(Gd,Nd)Mn_4_ phases precipitate at grain boundaries (GBs) during solidification. These diversified intermetallic compounds are beneficial for enhancing the mechanical properties of the alloys.

**Figure 4:**
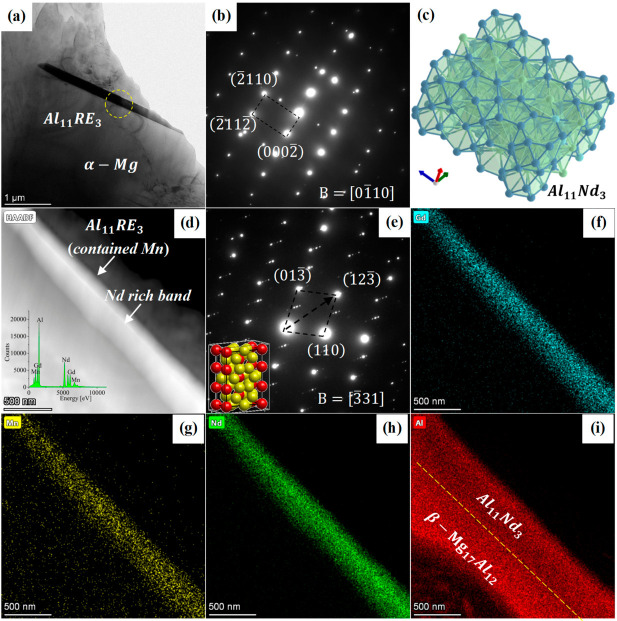
(**a**) TEM micrograph of Al_11_Nd_3_, (**b**), the corresponding SAED pattern, (**c**) 3D model of Al_11_Nd_3_, (**d**,**e**) HAADF image and the SAED patterns along $\left[\bar{3}31\right]$, (**f**–**i**) EDS mapping.

The detailed structures and formation mechanisms of primary intermetallic compounds formed during solidification were revealed by TEM. Figure 4a shows the bright-field TEM image of the rod-shaped phase with a diameter of 200 nm and a length of 3.0 μm in the 0.3Mn alloy. The phase is identified as Al_11_RE_3_ (orthorhombic structure, a = 4.36 Å, b = 9.97 Å, c = 12.96 Å) based on energy-dispersive spectroscopy (EDS), and the corresponding selected-area electron diffraction (SAED) pattern was taken from the $\left[01\bar{1}0\right]$ zone axis (Figure 4b). Figure 4c shows the simulated crystal structure of Al_11_RE_3_, which contributes to improving the creep resistance of Mg–Al–RE alloys. Figure 4d illustrates the high-angle annular dark-field image (HAADF) and the corresponding EDS spectrum of Al_11_(Nd,Gd)_3_; the Nd content is significantly higher than that of Gd with an atomic ratio of approximately 3:1. In addition, a Nd-rich band has been detected along the central symmetry axis of the Al_11_RE_3_ phase, suggesting that Nd atoms likely participate predominantly in the nucleation of the Al–Mn–RE phase, whereas Gd atoms incorporate mainly during subsequent growth as Nd becomes progressively depleted. The diffraction pattern in Figure 4e confirms that the Gd segregation does not alter the lattice parameters of Al_11_Nd_3_, and the final chemical formula is determined as Al_11_(Nd,Gd)_3_ [28]. It is worth noting that the presence of Mn in the Al_11_(Nd, Gd)_3_ is closely related to the Al–Mn–RE phase, which is a conclusion supported by the detection of two distinct sets of diffraction patterns in Figure 4e. Therefore, it can be inferred that the Al–Mn–RE phase serves as a heterogeneous nucleation site for Al_11_Nd_3_. Additionally, as shown in Figure 4f–i, it is noticeable that Al, Gd, and Mn are enriched in the rod-shaped particles.

**Figure 5:**
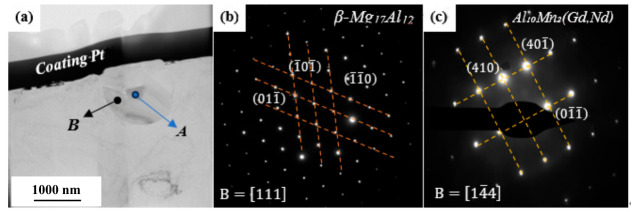
(**a**) TEM micrograph of intermetallic compounds, (**b**,**c**) the corresponding SAED pattern.

**Figure 6:**
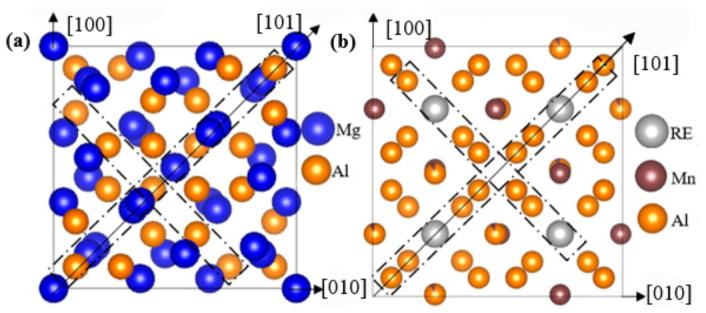
Distribution of Mg and Al atom arrangement along the [001] direction in different compounds (**a**) *β*-Mg_17_Al_12_; (**b**) Al_10_Mn_2_RE.

As can be seen from Figure 5a, the polygonal phase B is encapsulated at the center of the circular phase A. Based on transmission electron microscopy (TEM) and selected-area electron diffraction (SAED) (Figure 5b,c) analyses of the intermetallic compounds, they are identified as Al_10_Mn_2_(Gd,Nd) (phase A) and β-Mg_17_Al_12_ (phase B), respectively. It is worth noting that there is a low lattice mismatch (6.7%) between the *β*-Mg_17_Al_12_ phase (body-centered cubic structure) and the Al_10_Mn_2_(Gd,Nd) phase (body-centered tetragonal structure). Figure 6a,b shows the atomic arrangement of the two phases viewed along the [001] direction. The mismatch along the ${\left[101\right]}_{\beta }\;\mathrm{and}\;{\left[101\right]}_{{\mathrm{Al}}_{10}{\mathrm{Mn}}_{2}\mathrm{RE}}$ directions is 5.6%, and along the ${\left[\bar{1}0\bar{1}\right]}_{\beta }$ and ${\left[\bar{1}0\bar{1}\right]}_{{\mathrm{Al}}_{10}{\mathrm{Mn}}_{2}\mathrm{RE}}$ directions, it is 2.0%. The mismatch between the interplanar spacings of ${\left(100\right)}_{\beta }$ and ${\left(110\right)}_{{\mathrm{Al}}_{10}{\mathrm{Mn}}_{2}\mathrm{RE}}$ is 6.5%, and that between ${\left(111\right)}_{\beta }$ and ${\left(010\right)}_{{\mathrm{Al}}_{10}{\mathrm{Mn}}_{2}\mathrm{RE}}$ is 3.2%. The results indicate that Al_10_Mn_2_(Gd,Nd) satisfies the criteria to act as a heterogeneous nucleation site for the *β*-Mg_17_Al_12_ phase.

**Figure 7:**
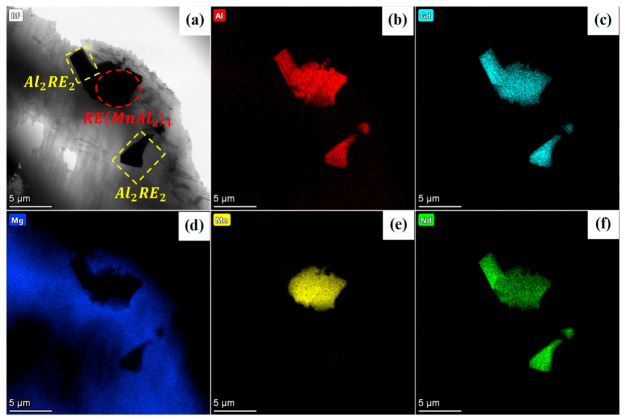
(**a**) BF-TEM image of precipitated intermetallics in 1.0Mn alloy, (**b**–**f**) EDS mapping of Mg, Al, Nd, Gd, Mn.

**Figure 8:**
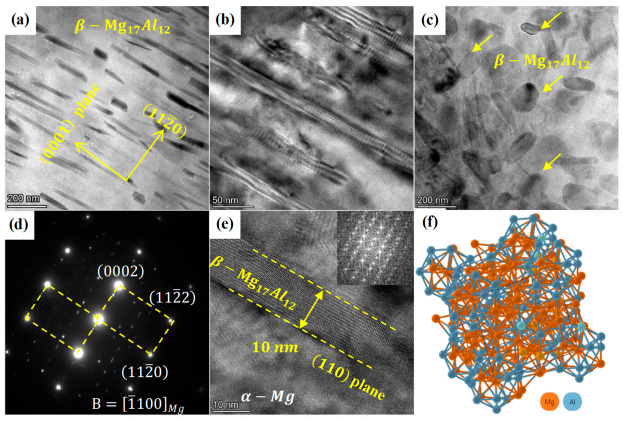
BF-TEM images of 1.0Mn alloy: (**a**,**b**) lath-shaped *β*-Mg_17_Al_12_ and corresponding enlarged image, (**c**) the granular *β*-Mg_17_Al_12_, (**d**) the SAED patterns along ${\left[\bar{1}100\right]}_{Mg}$, (**e**) HRTEM morphology of interface *β*/*α* matrix, the inserted FFT patterns in figure (**e**) is *β*, (**f**) 3D model of *β*-Mg_17_Al_12_.

Figure 7a presents the bright-field TEM image of blocky particles and Mn-containing particles. Based on the EDS analysis and the corresponding diffraction patterns, these two intermetallic compounds are identified as Al_2_RE (cubic structure; a = 7.89 Å) and Al_8_(Gd,Nd)Mn_4_ (tetragonal structure; a = 8.9 Å, b = 8.9 Å, c = 5.20 Å), respectively. The EDS results show that the Al_2_RE contains a low concentration of Mn (approximately 0.5 at. %), and the Gd content is significantly higher than that of Nd. Its unit-cell parameters are closer to those of the Al_2_Gd phase, as shown in Figure 7b–f. Figure 8a,b illustrate the bright-field (BF)TEM images of the lath-shaped precipitates with widths of 25–40 nm and lengths of 160–690 nm. In addition, spherical and short rod-shaped particles precipitated in the *α*-Mg matrix (Figure 8c), and the diameter of the spherical particles is about 100 nm. According to the corresponding SAED patterns (Figure 8d), the strip-shaped and spherical phases are verified as *β*-Mg_17_Al_12_ (body-centered cubic structure; a = 1.056 nm) [29]. It is worth noting that the lath-shaped and spherical *β*-Mg_17_Al_12_ exhibit morphology similar to that of continuously precipitated *β*-Mg_17_Al_12_ during aging, which may be attributed to Mn addition increasing the solid solubility of Al atoms in the α-Mg matrix, thereby promoting the precipitation of excess Al atoms, resulting in the precipitation of excess Al atoms during the cooling process. These nanoscale *β*-Mg_17_Al_12_ precipitates play an important role in enhancing the tensile strength of the alloy. Figure 8e exhibits the HRTEM image and the corresponding FFT patterns of the *β*/*α* interface; it is revealed that the parallel lath-shaped *β*-Mg_17_Al_12_ (the crystal cell structure is shown in Figure 8e) precipitates along the (0001) plane of the *α*-Mg matrix, maintaining a Burgers orientation relationship. Additionally, the habit plane of the lath-shaped *β*-Mg_17_Al_12_ deviates from the (0001)_Mg_ base plane by approximately 12.91°, i.e., ${\left(110\right)}_{\beta }//\;{\mathrm{from}\;12.91\text{°}\left(0001\right)}_{\mathrm{Mg}}$.

**Figure 9:**
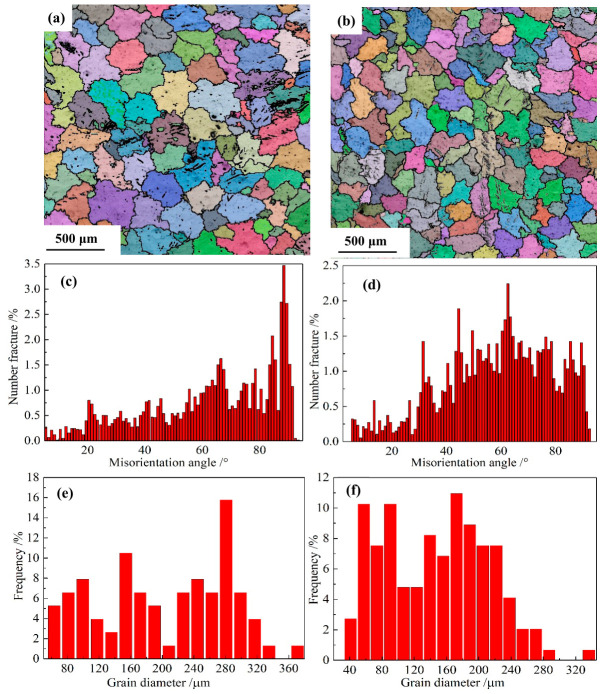
EBSD maps with all Euler of as-cast alloys: (**a**) 0.3 wt. % Mn, (**b**) 1.0 wt. % Mn, (**c**,**d**) misorientation angle distributions, (**e**,**f**) and the corresponding grain size distribution.

Figure 9a,b shows the grain orientation maps of 0.3Mn and 1.0Mn alloys. The grain structure of the as-cast Mn-containing alloys exhibits a random orientation and shows no obvious texture characteristics. According to the misorientation angle distribution maps in Figure 9c,d, the microstructure of the 0.3Mn alloy has low-angle grain boundaries (LAGBs) accounting for 2.1% of all grain boundaries, whereas the majority (97.9%) are high-angle grain boundaries (HAGBs). Compared with the 0.3 wt. % Mn-containing alloy, the proportion of LAGBs in the 1.0Mn specimen increases to 4.2%, which may be related to the formation of dislocation substructures. Relevant research has shown that LAGB structures are not easily moved or prone to slip, thereby hindering grain boundary migration to some extent [30]. Figure 9e,f illustrate that the grain size of *α*-Mg matrix ranges from 160 to 280 μm, yielding average values of approximately 197 μm (for 0.3 wt. % Mn) and 152 μm (for 1.0 wt. % Mn). Compared with our previous results on the Mg–8Al–1.0Nd–1.5Gd alloy (grain size ≈ 300 μm), it can be seen that the trace addition of Mn leads to an excellent grain refinement effect.

### 3.2. Creep Behavior and Mechanical Property

**Figure 10:**
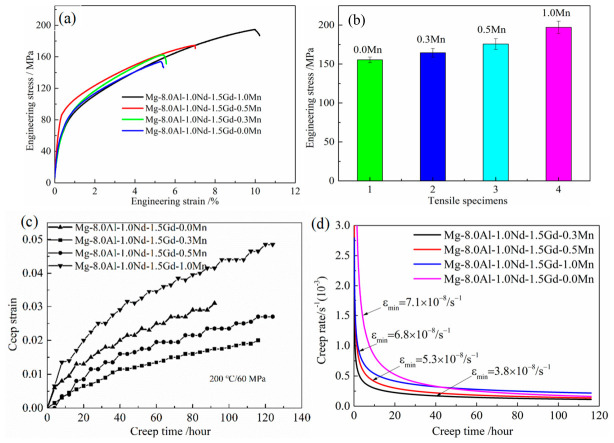
(**a**) Stress–strain curve [31], (**b**) tensile strength corresponding to different alloys, (**c**) the creep strain curves and (**d**) the corresponding fitted minimum creep rates.

Figure 10a,b show the tensile stress–strain curves and the tensile strength with standard deviation. It can be seen that the 1.0Mn alloy exhibits the highest tensile strength and elongation, with values of 194.6 MPa and 9.8%, respectively. Compared with the Mn-free alloy, the tensile strength increases by 26.2% and the elongation by 92.1% [31]. As illustrated in Figure 10c, representative creep curves for the investigated alloys were obtained at 200 °C under a stress of 60 MPa; the minimum creep rate ($\dot{\varepsilon }$, Figure 10d) was determined from the slope of the linear fit to the steady-state region of the creep curve. It is apparent that the alloy with 0.3 wt. % Mn exhibits optimal creep resistance with a minimum creep rate of $3.8\;\text{×}\;10^{-8}/s^{-1}$. Although the creep resistance slightly decreases as the Mn content increases to 1.0 wt. %, the high-temperature creep performance remains superior to that of Mn-free alloy, and it also maintains excellent tensile strength and elongation.

**Figure 11:**
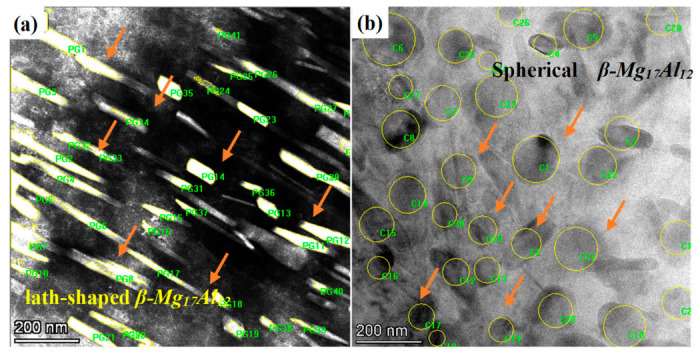
Parameter measurement for the Orowan increment in CRSS of 1.0Mn alloy: (**a**) lath-shaped *β*-Mg_17_Al_12_ precipitates, (**b**) spherical *β*-Mg_17_Al_12_ precipitates.

The Mn-added alloy exhibits excellent mechanical properties, which have been discussed in our previous study and are primarily attributed to two factors: (1) strengthening from second-phase particles generated by intermetallic compounds, and (2) grain refinement strengthening resulting from the refined *α*-Mg matrix grains [31]. It should also be noted that the nanoscale *β*-Mg_17_Al_12_ present in the as-cast microstructure also contributes an Orowan strengthening increment, which enhances the superior tensile strength of the 1.0Mn alloy. Based on the structural characterization of the alloy described above (Figure 11a,b), a large number of lamellar and ellipsoidal primary *β*-Mg_17_Al_12_ with a dispersed distribution are observed. Using Image-Pro Plus to calculate the phase volume fraction, the area fraction of the *β*-Mg_17_Al_12_ phase measured from transmission electron microscopy (TEM) images can be statistically approximated as the volume fraction of that phase within the material.

According to the Hall–Petch relationship [19,32],

(1) $${\text{∆}\sigma }_{\mathrm{ys}}=kd^{-1/2}$$

where $\delta_{ys}$ is the yield stress, *k* is the Hall–Petch slope and *d* is the average grain diameter. The grain refinement can play a key role in improving the tensile strength and elongation of Mn-contained Mg–8.0Al–1.0Nd–1.5Gd alloys. The grain boundary increases significantly due to grain refinement, resulting in uniform grain deformation and hindering the propagation of microcracks caused by stress concentration [33].

In addition, the dispersion strengthening caused by refined intermetallic compounds enhances tensile strength, which is known as the Orowan mechanism [34,35]. The Orowan increment ∆τ in CRSS induced by the obstruction of dislocation motion is shown in Equation (2):

(2) $$\text{∆}\tau \;=\;\frac{\mathrm{Gb}}{2\pi \lambda \sqrt{1-\upsilon }}\ln\frac{d_{p}}{r_{0}}$$

where *G* is the shear modulus (17 GPa), *b* is the Burgers vector, *υ* is the Poisson ratio (0.29), *λ* is the effective inter-particle spacing, $d_{p}$ is the average radius of the *β*-Mg_17_Al_12_, and $r_{0}$ is the core radius of dislocations. Based on spherical and lath-shaped precipitates along the (0001)_Mg_ plane, the revised Orowan increment is characterized as Equations (3) and (4), respectively [36].

(3) $${\Delta \tau }_{\mathrm{spherical}}=\frac{\mathrm{Gb}}{2\pi d_{t}\sqrt{1-\upsilon }\left(\frac{0.953}{\sqrt{f}}-1\right)}\ln\frac{d_{t}}{b}$$

(4) $${\Delta \tau }_{\mathrm{lath}}=\frac{\mathrm{Gb}}{2\pi d_{t}\sqrt{1-\upsilon }\left(\frac{0.779}{\sqrt{f}}-0.785\right)}\ln\frac{{0.785d}_{t}}{b}$$

where *f* is the volume fraction of *β*-Mg_17_Al_12_, two sets of parameters ($d_{t}\;=\;97.2\;\mathrm{nm}$, *f* = 0.23 and $d_{t}\;=\;193.6\;\mathrm{nm}$, *f* = 0.2) are substituted into Equations (2) and (3), respectively (Figure 9), and the Orowan increment was calculated to be ${\Delta \tau }_{\mathrm{spherical}}\;=\;32.6\;\mathrm{MPa}$, ${\Delta \tau }_{\mathrm{lath}}\;=\;17.9\;\mathrm{MPa}$. The result indicates that the yield stress produced by spherical particles is superior to those produced by lath precipitates.

### 3.3. Characterization of Morphological Evolution of Crept Specimens

**Figure 12:**
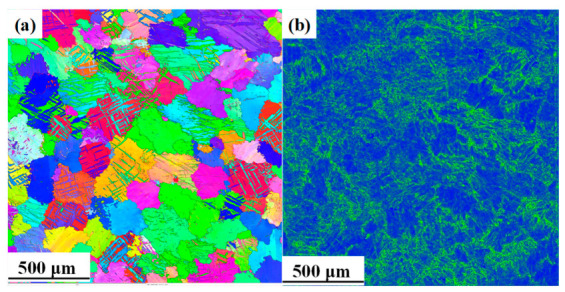
The EBSD map with all Euler of the 0.3Mn alloy crept at 200 °C/60 MPa: (**a**) Orientation Imaging Map; (**b**) Kernel Average Misorientation (KAM).

**Figure 13:**
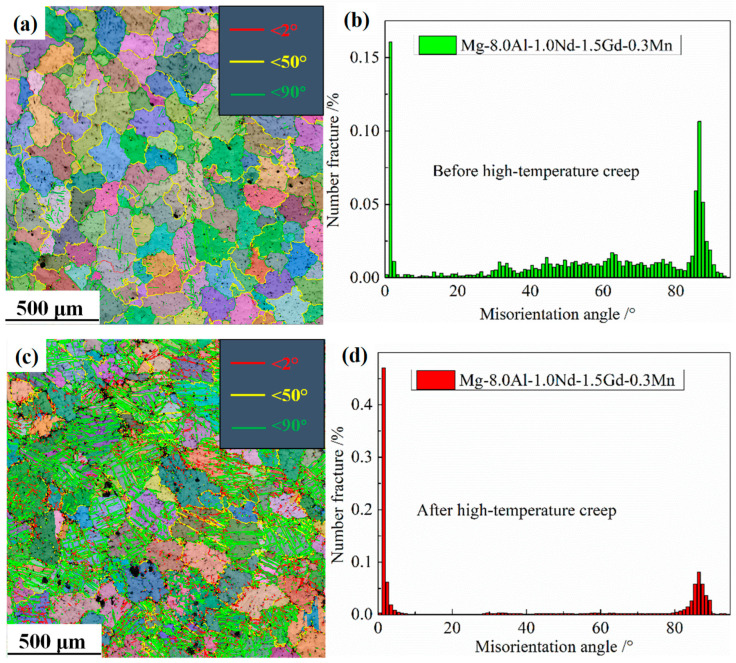
Distribution characteristics of grain boundary angles and the corresponding misorientation angle distribution maps in deformed grains of 0.3Mn alloy: (**a**,**b**) before creep deformation; (**c**,**d**) after creep deformation.

According to the creep curves, the 0.3Mn alloy exhibits the optimal creep performance. However, as the Mn content increases to 1.0 wt. %, the creep performance begins to deteriorate, necessitating further in-depth characterization. Figure 12a,b presents the OIM (Orientation Imaging Map) and KAM (Kernel Average Misorientation) of the 0.3Mn alloy after creep deformation. It can be observed that the creep microstructure contains a large number of tensile twins, which accommodate deformation along the c-axis. The KAM reveals significant stress concentration at the *α*-Mg grain boundaries during creep, suggesting the occurrence of grain boundary sliding (GBS) during deformation. Additionally, combined with the grain boundary misorientation angle distribution and grain misorientation statistics of the 0.3Mn alloy before and after creep shown in Figure 13, it is found that high-angle grain boundaries (HAGBs) dominate the pre-creep microstructure, whereas a substantial number of low-angle grain boundaries (LAGBs) are generated after creep; the misorientation angle distribution is concentrated at ~86, indicating the presence of $86.3\text{°}\left\{10\bar{1}2\right\}$ tensile twins, and the LAGBs (<5°) account for 60% of all grain boundaries—consistent with subgrain boundaries induced by extensive dislocation slip [37]. This further confirms the significant activation of grain boundary sliding during creep. With grain refinement, the contribution of grain boundary sliding to creep deformation increases, which is a crucial factor responsible for the degradation of creep performance in the 1.0Mn alloy. The identification and formation mechanisms of tensile twin variants have been discussed in our previous research. Tensile twins can coordinate the slip deformation between adjacent grains, improving the strain compatibility between adjacent grains by twinning transfer across the grain boundary [38,39].

**Figure 14:**
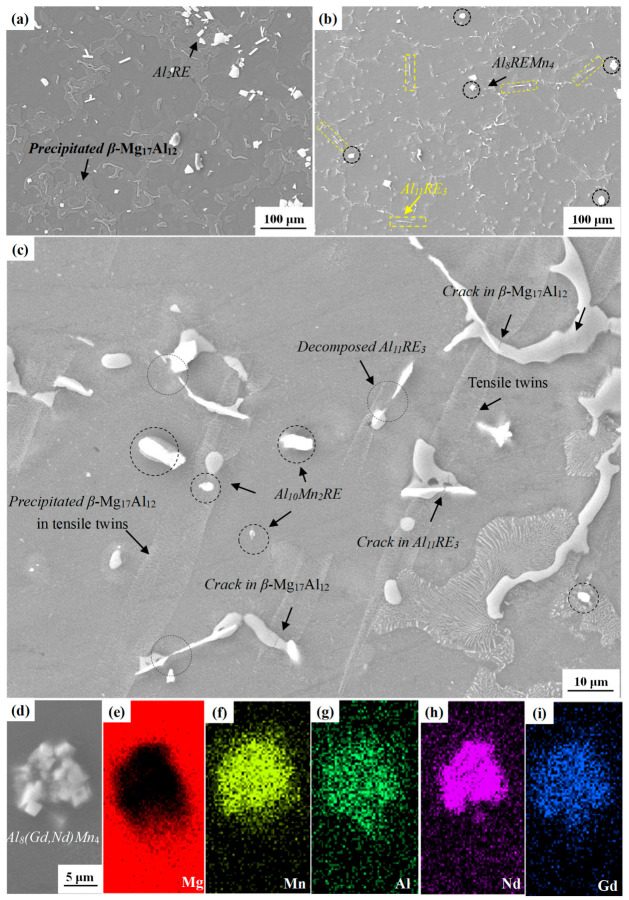
Morphology of Mg–8.0Al–1.5Gd–1.0Nd–0.3Mn alloy after being crept at 200 °C/60 MPa for 100 h: (**a**) Mn-free alloy, (**b**,**c**) 1.0Mn alloys and corresponding magnified images, (**d**) SEM image of Al_8_(Gd,Nd)Mn_4_, and corresponding EDS mapping (**e**) Mg; (**f**) Mn; (**g**) Al; (**h**) Nd; (**i**) Gd.

Figure 14a,b show the SEM micrographs of the Mg–8.0Al–1.5Gd–1.0Nd–0.3Mn alloy after creep testing; it can be seen that the content of *β*-Mg_17_Al_12_ precipitated at the grain boundaries of 0.3Mn alloy is lower than that in the Mn-free alloy. Moreover, microcracks are mainly concentrated in coarse eutectic *β*-Mg_17_Al_12_ (Figure 14c), which decreases the creep resistance of the alloy. In addition, no significant change is observed in Al_2_(Gd,Nd) after creep, which is attributed to its excellent thermal stability. In contrast, Al_11_(Gd,Nd)_3_ undergoes partial decomposition under creep stress, leading to microcrack formation. It is worth noting that the Al_8_(Gd,Nd)Mn_4_ and Al_12_(Gd,Nd)_2_Mn_5_ phases in the 0.3Mn alloy substitute for Al_2_(Gd,Nd) and still exhibit outstanding high-temperature creep resistance. As shown in Figure 14d–i, the Al_8_(Gd,Nd)Mn_4_ maintains excellent thermal stability during creep at 200 °C/60 MPa—a finding of great significance for improving the creep performance of Mg–8.0Al–1.0Nd–1.5Gd–*x*Mn alloys.

**Figure 15:**
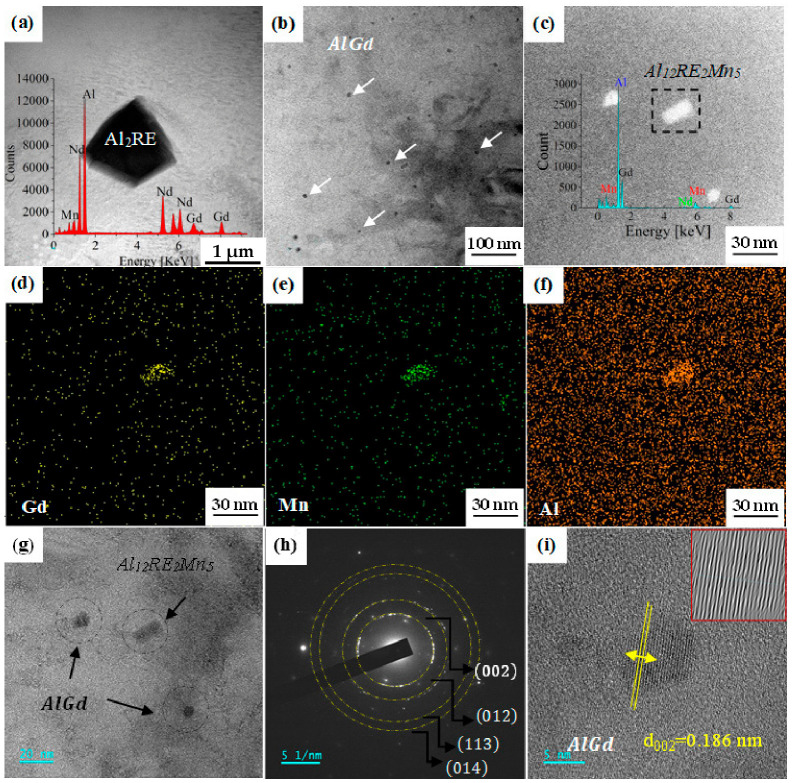
BF-TEM images of Mg–8.0Al–1.5Gd–1.0Nd–0.3Mn alloy after being crept at 200 °C/60 MPa for 100 h: (**a**,**b**) Al_2_RE and Al_12_RE_2_Mn_5_, (**c**) spot spectrum of Al_12_RE_2_Mn_5_ and (**d**–**f**) corresponding EDS mapping; (**g**,**h**) the nanoscale spherical precipitates and the corresponding SAED pattern, (**i**) HRTEM of the AlGd phase; the insert is the corresponding IFFT patterns.

**Figure 16:**
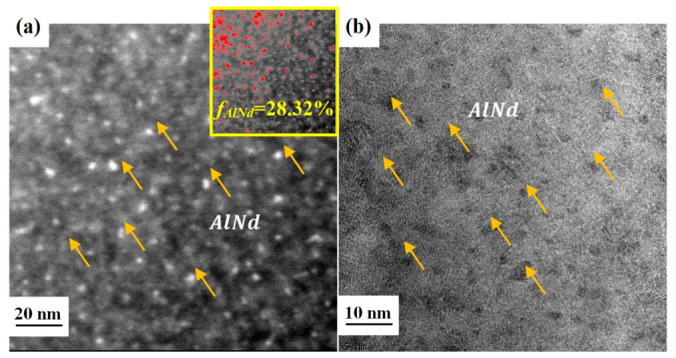
(**a**)The STEM-HAADF image of the AlNd precipitate in the *α*-Mg matrix of 0.3Mn alloy and (**b**) the corresponding HRTEM image.

Figure 15a–c display the STEM/EDS analysis of the intermetallic phases in the 0.3Mn alloy after creep testing at 200 °C/60 MPa for 100 h. The typical Al_2_RE values (approximately 1–2 μm) with excellent thermal stability are conducive to enhancing creep performance [6]. Figure 15d–f show the STEM/EDS elemental mapping of Al, Mn, and Gd; the rectangular precipitate phase (approximately 20 nm) with a composition of Al_12.5_Mn_5.06_Nd_0.54_Gd_1.14_ exhibits an atomic ratio close to Al_12_RE_2_Mn_5_. Figure 15g displays the micrographs of the dynamically precipitated phases in the specimen crept at 200 °C/60 MPa for 100 h; the diameter of nanoscale spherical precipitates ranges from 5 to 10 nm and exhibits a dispersed distribution. As shown in Figure 15h, the precipitates are identified as the AlGd phase (orthorhombic structure, a = 7.48 Å, b = 9.17 Å, c = 5.59 Å) based on the corresponding SAED pattern. Figure 15i illustrates the HRTEM morphology with the FFT patterns of the dynamically precipitated AlGd phase; the measured interplanar spacing of the (002) plane is 0.186 nm. Meanwhile, a large number of dynamically precipitated AlNd particles (as shown in Figure 16) with a size of approximately 1–2 nm were observed within the magnesium matrix, and the volume fraction of the precipitated AlNd phase is approximately 28.32%. These particles exhibit good thermal stability, enabling them to pin dislocations and thereby improve the creep resistance of the alloy. The crystallographic orientation relationship and precipitation mechanism of the AlGd phase need elaborate efforts to be revealed.

The addition of Mn promotes the formation of Al–Mn–RE intermetallic phases with superior creep resistance compared to those in Mn-free alloys. Table 1 lists the formation energies of the corresponding intermetallics; it can be concluded that these intermetallics possess significantly lower formation energies than that of the *β*-Mg_17_Al_12_ phase (−0.027 eV/atom), thereby exhibiting exceptional thermal stability [40,41]. In addition, nanoscale spherical precipitates play an important role in elevating the creep performance of the alloys.

**Figure 17:**
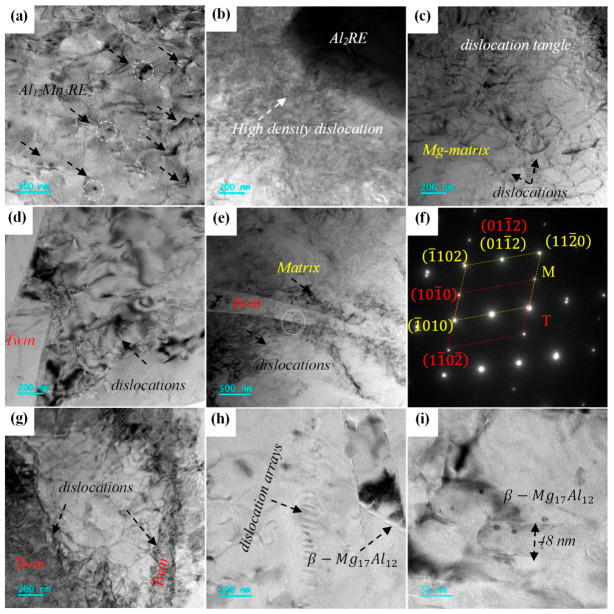
Dislocation configurations in the crept 0.3Mn specimen: (**a**) fine Al_12_Mn_5_RE_2_ particles, (**b**) dislocations accumulated at Al_2_RE, (**c**) dislocation structure in Mg matrix, (**d**,**e**) extension twining in the grain interior and (**f**) corresponding SAED patterns viewed with B = [$0\bar{2}$1], (**g**) interaction between twin boundaries and dislocations, (**h**) dislocation array formed during creep, (**i**) the coarsened *β*-Mg_17_Al_12_.

Figure 17 reveals the microstructural evolution of the 0.3Mn alloy after creep testing. The excellent creep resistance is mainly attributed to the presence of multiple intermetallic phases. Blocky Al_2_Gd, acicular Al_11_Nd_3_, and Al_8_Mn_4_RE particles can effectively pin grain boundaries (GBs) and suppress dislocation slip at elevated temperatures [42]. Simultaneously, Al_12_Mn_5_RE_2_ particles dispersed within the grains hinder dislocation movement and improve high-temperature creep resistance. The Al_12_Mn_5_RE_2_ phase is structurally similar to Al_12_Sm_2_Mn_5_, which significantly improves the ductility of the ASm44 alloy [43]. Figure 17a reveals the bright-field TEM images of dislocation substructures in the crept specimen; the Al_12_Mn_5_RE_2_ phase exhibits excellent thermal stability and efficiently impedes dislocation motion. In addition, GBs decorated with the typical Al_2_Gd phase were also observed in the crept 0.3Mn alloy with substantial dislocations piling up (Figure 17b) at the interface of the *α*-Mg matrix. Figure 17c illustrates significant bending as dislocations bypass second-phase particles. The activated slip modes in the crept specimen are dominated by basal slip, and <*c + a*> dislocations are also activated. Additionally, tensile twins with a typical lenticular shape are observed in the Mg–8.0Al–1.5Gd–0.3Mn alloy after creep testing at 200 °C/60 MPa for 100 h (Figure 17d,e). The corresponding SAED pattern of the twin boundary region (marked by the white circle in Figure 17f) shows mirror diffraction spots associated with the $\left(01\bar{1}2\right)$ plane, indicating that the twins are $\left\{10\bar{1}2\right\}$ with a misorientation angle of 86° [44]. It is worth noting that the twin boundaries act as obstacles to gliding dislocation, leading to dislocation pile up at these boundaries. As shown in Figure 17g, a high density of dislocations is observed inside the twins, indicating that twinning induces a significant crystallographic orientation deviation—thereby facilitating dislocation slip [45]. In twin-free regions of the α-Mg grain interior, dislocations are homogeneously distributed, whereas dislocation pile-up is evident near twin boundaries [46,47]. Experimental results reveal that the dominant creep deformation is governed by dislocation slipping and twin deformation. Figure 17h shows dislocation arrays on the slip planes of the *α*-Mg matrix; these dislocations eventually accumulate at grain boundaries, forming subgrain boundary structures. Meanwhile, the grain boundaries of *β*-Mg_17_Al_12_ provides obstacles for dislocation slip. Figure 17i shows that the as-cast *β*-Mg_17_Al_12_ has coarsened; these microstructural evolutions deteriorate the creep resistance of the alloy [48].

## 4. Discussion

Compared with the Mg–8.0Al–1.0Nd–1.5Gd alloy, the Mg–8.0Al–1.0Nd–1.5Gd–-*x*Mn (*x* = 0.0, 0.3, 0.5, 1.0 wt. %) alloy exhibits a precipitation of intermetallic compounds including, in addition to the common Al_2_RE and Al_11_RE_3_ phases, ternary Al–Mn–RE phases—such as Al_8_(Gd,Nd)Mn_4_ and Al_10_Mn_2_(Gd,Nd)—induced by the addition of a trace amount of Mn. Meanwhile, the grains of the *α*-Mg matrix and the *β*-Mg_17_Al_12_ phase are significantly refined, and the intermetallic compounds exhibit a dispersed distribution along the grain boundaries and within the *α*-Mg matrix grains. Previous studies attributed the refinement of the α-Mg matrix and the *β*-Mg_17_Al_12_ phase primarily to the heterogeneous nucleation sites provided by Al_8_Mn_4_ [13,49]. However, this paper reveals that Al_10_Mn_2_(Gd,Nd) also serves as a heterogeneous nucleation site for *β*-Mg_17_Al_12_, promoting the transformation of the eutectic *β*-Mg_17_Al_12_ from a coarse network morphology into a granular form. This is the main reason why the Mg–8.0Al–1.0Nd–1.5Gd–xMn alloy exhibits excellent mechanical properties.

Regarding high-temperature creep resistance, the 0.3Mn alloy shows the best creep resistance. With increasing Mn content, the creep performance of the 1.0Mn alloy begins to decrease, but its minimum creep rate remains essentially at the same level as that of the Mg–8.0Al–1.0Nd–1.5Gd alloy. We have significantly improved the elongation and tensile strength of the alloy while maintaining the excellent creep resistance of the Mn-free alloy, which is one of the key innovations of this paper. A microstructural analysis of the crept specimens reveals that thermally stable Al_8_REMn_4_ and Al_10_Mn_2_(Gd,Nd) hinder dislocation slip during creep. Concurrently, nano-sized AlGd and AlNd dynamically precipitated within the *α*-Mg matrix also effectively impede dislocation slip, thereby enhancing the creep deformation resistance. Although these precipitates are similar in strengthening effect to the Al–Y–Mn cluster phases reported in Mg–8.44Y–0.9Al–0.6Mn alloy [50], the dynamic precipitated AlRE phases were discovered to be innovative in the Mg–Al–RE alloys. These intermetallic compounds effectively mitigate the negative impact of grain refinement in the *α*-Mg matrix on creep performance, enabling the Mg–8.0Al–1.0Nd–1.5Gd–*x*Mn alloy to possess both excellent elongation and high creep resistance.

## 5. Conclusions

The precipitation characteristics of intermetallic compounds and creep evolution behavior of the Mg–8.0Al–1.0Nd–1.5Gd-*x*Mn(*x* = 0.0, 0.3, 0.5, 1.0 wt. %) alloy were thoroughly investigated. The addition of trace amounts of Mn promotes the dispersed precipitation of Al–RE–Mn phases at the grain boundaries of the *α*-Mg matrix (the volume fraction of intermetallics compound (*f_IMC_*) increased from 6.86% to 12.24%). The Al_10_Mn_2_(Gd,Nd) can act as a heterogeneous nucleation site for the *β*-Mg_17_Al_12_, resulting in significant grain refinement. Micron-scale thermally stable Al_8_(Gd,Nd)Mn_4_ and Al_10_Mn_2_(Gd,Nd) particles, as well as a nanoscale AlNd/Gd cluster, were introduced into Mn-containing Mg–8.0Al–1.0Nd–1.5Gd alloy to enhance creep resistance. Thereby, the negative impact of the increased proportion of grain boundary sliding caused by *α*-Mg grain refinement has been balanced. The as-cast Mg–8.0Al–1.0Nd–1.5Gd–*x*Mn alloy exhibits an excellent elongation of 9.8% while maintaining a low creep rate of $3.8\;\text{×}\;10^{-8}/s^{-1}$ under 200 °C and 50 MPa. This is of great significance for the development and design of Mg–Al–RE alloys combining excellent creep resistance and plastic deformation capability.

## Figures and Tables

**Table 1:** Formation energies of the intermetallic compounds in Mg–8.0Al–1.0Nd–1.5Gd–*x*Mn alloys.

Intermetallic Phases	Al_2_Gd	Al_8_Mn_4_RE	AlGd	Al_11_RE_3_	Al_12_Mn_5_RE_2_
Formation Energy (eV/atom)	−0.46	−0.32	−0.41	−0.37	−2.17

## Data Availability

The original contributions presented in this study are included in the article. Further inquiries can be directed to the corresponding author.
